# Carbidopa, an activator of aryl hydrocarbon receptor, suppresses IDO1 expression in pancreatic cancer and decreases tumor growth

**DOI:** 10.1042/BCJ20210851

**Published:** 2022-09-06

**Authors:** Ksenija Korac, Devaraja Rajasekaran, Tyler Sniegowski, Bradley K. Schniers, Andrew F. Ibrahim, Yangzom D. Bhutia

**Affiliations:** 1 Department of Cell Biology and Biochemistry, Texas Tech University Health Sciences Center, Lubbock, TX 79430, U.S.A.; 2 Department of Biological Sciences, Texas Tech University, Lubbock, TX 79410, U.S.A.

**Keywords:** aryl hydrocarbon receptor, carbidopa, indoleamine dioxygenase 1, JAK/STAT signaling, pancreatic cancer

## Abstract

IDO1 is an immunomodulatory enzyme responsible for tryptophan catabolism. Its expression in immune cells, especially the DCs, has attracted attention because it leads to tryptophan depletion at the immunological synapse, thereby causing T-cell anergy and immune evasion by the tumor cells. Cancer cells also overexpress IDO1. Immunotherapy targeting IDO1 has been one of the focus areas in cancer biology, but lately studies have identified non-immune related functions of IDO1 leading to a paradigm shift with regard to IDO1 function in the context of tumor cells. In this study, we show that PDAC tissues and PDAC cells overexpress IDO1. The expression level is reciprocally related to overall patient survival. We further show that carbidopa, an FDA-approved drug for Parkinson's disease as well as an AhR agonist, inhibits IDO1 expression in PDAC cells. Using athymic nude mice, we demonstrate that carbidopa-mediated suppression of IDO1 expression attenuates tumor growth. Mechanistically, we show that AhR is responsible for carbidopa-mediated suppression of IDO1, directly as a transcription factor and indirectly by interfering with the JAK/STAT pathway. Overall, targeting IDO1 not only in immune cells but also in cancer cells could be a beneficial therapeutic strategy for PDAC and potentially for other cancers as well and that carbidopa could be repurposed to treat cancers that overexpress IDO1.

## Introduction

IDO1, an immunomodulatory tryptophan-catabolizing enzyme [1], is expressed in several normal tissues like the lymph node, spleen, thymus, lungs, epididymis, colon, and at the maternal-fetal interface wherein it is known to play a vital role in regulating local and peripheral immune tolerance [2–6]. It is highly up-regulated in tumor cells, in tumor-surrounding stroma, which is composed of endothelial cells, immune cells, fibroblast, and mesenchymal cells, and in tumor-draining lymph nodes. Expression of IDO1 in tumor cells is associated with significantly worse clinical prognosis and reduced overall survival in many cancer types including ovarian cancer [7], malignant melanoma [8,9], pediatric [10] and adult acute myelogenous leukemia [11,12], colorectal cancer [13], prostate cancer [14], endometrial cancer [15], as well as in pancreatic cancer [16]. It is shown that the increased IDO1 activity depletes Trp while increasing kynurenine concentration in the microenvironment surrounding the IDO1-expressing cells and that the resultant Trp depletion and kynurenine production synergistically reduce effector T cell proliferation and promote differentiation of immunosuppressive T-regulatory cells [17–21]. This creates an immunologically favorable environment for tumor growth by facilitating immune evasion by tumor cells.

Over the last several years, there has been a significant interest in targeting IDO1 for cancer therapy. However, this therapeutic approach has been investigated predominantly in the context of immune regulation and immunotherapy. Several IDO1 inhibitors have been identified and some have advanced to clinical trials and proven efficacious at least in combination with other chemotherapeutic agents. The fact that Trp depletion activates GCN2 and consequently inhibits mTORC1 signaling pathway is the idea behind why IDO1 overexpression leads to T-cell anergy. 1-MT is an inhibitor of IDO1, but the active isomer for this inhibition is the L-form, not the D-form. However, the D-isomer (D-1MT) has proven efficacious *in vivo* in blocking tumor growth; it seems that D-1MT has the ability to mimic Trp and provides Trp sufficiency signal for T-cell growth [22]. While the incorporation of immunotherapy in the treatment algorithm of many solid tumors has marked a therapeutic renaissance, its effect on PDAC has been uniformly disappointing. The intense desmoplastic stroma could be the cause for the low immunogenicity of PDAC [23]. Interestingly, there has been more focus in recent years on targeting IDO1 in the cancer cells than in immune cells. Studies have revealed that cancer cells specifically up-regulate IDO1 with the purpose of utilizing Trp metabolites as signaling molecules and also as a source of one-carbon donor for purine nucleotide synthesis for their proliferation [24]. This suggests that apart from its immune regulatory function, IDO1 seems to have non-immune related functions as well. Therefore, targeting IDO1 in tumor cells could be a novel therapeutic strategy for IDO1-expressing tumors.

Carbidopa is an FDA approved drug that is used with L-DOPA to treat Parkinson's disease. However, it is not used by itself for any indication. Parkinson's disease patients exhibit lower incidence of most cancers including pancreatic cancer, but with the notable exception of melanoma [25–28]. The decreased cancer incidence is not due to L-DOPA, leaving the possibility that carbidopa might have anti-cancer efficacy. Our published studies have established the anti-cancer efficacy of carbidopa in pancreatic cancer and also have discovered that carbidopa is an agonist for the nuclear receptor AhR [29]. Based on structural similarity with phenylhydrazine, an inhibitor of IDO1, we predicted that carbidopa might also inhibit IDO1. Our preliminary investigations did suggest its ability to inhibit IDO1 directly [29]. However, with the new discovery that carbidopa activates AhR, we asked if this drug would interfere with IDO1 expression, thus potentially using another mechanism for suppression of IDO1 activity. If this proves to be true, carbidopa could potentially be repurposed to treat PDAC and be fast-tracked into clinic for cancer therapy either as a single agent or in combination with other standard chemotherapeutic agents.

In the present study, we provide evidence that carbidopa does indeed suppress the expression of IDO1 in PDAC cells and that this process is mediated by carbidopa-dependent activation of AhR. The suppressive effect of carbidopa is seen not only with the constitutively expressed IDO1 but also with IFN-γ-induced IDO1 expression, demonstrable both at the mRNA level and the protein level. Carbidopa elicits this effect via AhR, which suppresses IDO1 expression by two independent mechanisms, first by the direct action as a transcription factor with IDO1 as its target and second by the indirect action with the blockade of the JAK/STAT pathway. We also demonstrate the anti-cancer effect on PDAC using an *in vivo* xenograft mouse model and provide evidence that the observed anticancer effect is IDO1-mediated. Taken together, our study provides proof that targeting IDO1 in tumor cells can be a beneficial therapeutic approach for PDAC and expands the therapeutic applications of carbidopa beyond Parkinson's disease to target IDO1 in cancers.

## Results

### IDO1 is up-regulated in PDAC and affects overall patient survival

**Figure 1.**
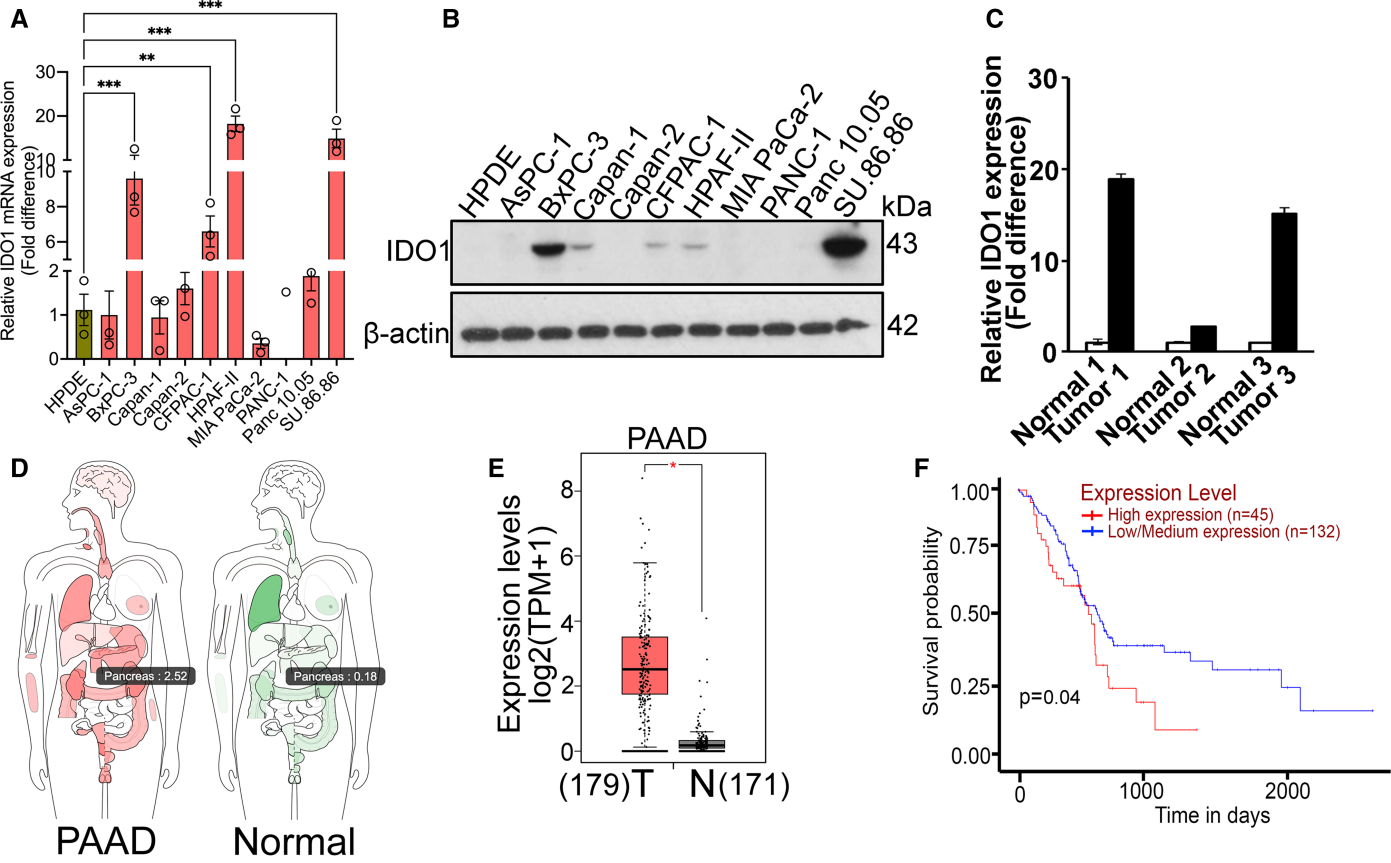
IDO1 is up-regulated in PDAC and affects overall patient survival. (**A**) Real-time PCR showing relative IDO1 mRNA expression in HPDE cell line and 10 PDAC cell lines. (**B**) Western blotting showing IDO1 protein expression in normal pancreatic cell line (HPDE) and 10 PDAC cell lines. β-actin was used as an endogenous control. (**C**) Real-Time PCR showing relative IDO1 mRNA expression in matched PDAC primary tumors vs. the corresponding normal pancreas. (**D**) Interactive body map showing IDO1 expression in normal tissues and pancreatic adenocarcinoma (PAAD). (**E**) Box plot map showing TPM-normalized expression of IDO1 in normal tissues and pancreatic adenocarcinoma (PAAD). Since there are only four normal pancreatic samples in TCGA data, we combined them with the pancreatic samples in GTEx data. The results in D & E were generated by the online tool GEPIA (http://gepia.cancer-pku.cn/index.html). (**F**) Kaplan–Meier plot showing survival probability between high and low IDO1 expression in pancreatic cancer based on TCGA database. The result was generated from the online tool UALCAN (http://ualcan.path.uab.edu). Significance of survival impact is measured by log-rank test. For all *in vitro* studies *n* = 3. Data are means ± SEM. Statistical significance for (**A**) was assessed using one-way ANOVA followed by Dunnett's *post hoc* test whereas for (**E**), one-way ANOVA using tumor or normal as variable was used for calculating differential expression (* *P* < 0.05; ** *P* < 0.01; *** *P* < 0.001). T, tumor; N, normal.

To determine the relevance of IDO1 that is expressed in cancer cells in PDAC, we first analyzed IDO1 mRNA by real-time RT-qPCR in 10 PDAC cell lines (AsPC-1, BxPC-3, Capan-1, Capan-2, CFPAC-1, HPAF-II, MIA PaCa-2, PANC-1, Panc 10.05, and SU.86.86) and compared the levels to that in HPDE, a normal pancreatic ductal epithelial cell line. Interestingly, many PDAC cell lines like BxPC-3, CFPAC-1, HPAF-II, and SU.86.86 showed a significant up-regulation of IDO1 mRNA as opposed to the normal HPDE (Figure 1A). IDO1 protein expression as shown by Western blotting also corroborated with the mRNA data (Figure 1B). Next, we checked IDO1 mRNA expression in three sets of matched PDAC primary tumors vs. the corresponding normal pancreas and here too, the tumor samples clearly showed a significant up-regulation of IDO1 mRNA as opposed to normal pancreas (Figure 1C). To further corroborate these data from cell lines and primary tumors, we used the web-based tool GEPIA (Gene Expression profiling Interactive Analysis) and checked the expression profile of IDO1 in PDAC tumor vs. normal pancreas. The interactive body map as well as the box plot describing quantitatively the expression levels clearly showed significant up-regulation of IDO1 expression in PDAC tumors as opposed to normal pancreas (Figure 1D,E). To determine if the IDO1 expression has any relation to disease severity, we analyzed the data from the Cancer Genome Atlas (TCGA). This analysis showed a reciprocal relationship between IDO1 expression levels and survival probability; the higher the IDO1 expression, the lower is the survival probability in PDAC patients (Figure 1F). Collectively, these data indicate that IDO1 is up-regulated in PDAC tumors and PDAC cancer cells and has an impact on overall patient survival.

### Carbidopa decreases IDO1 mRNA in PDAC cells with corresponding change in IDO1 protein

**Figure 2.**
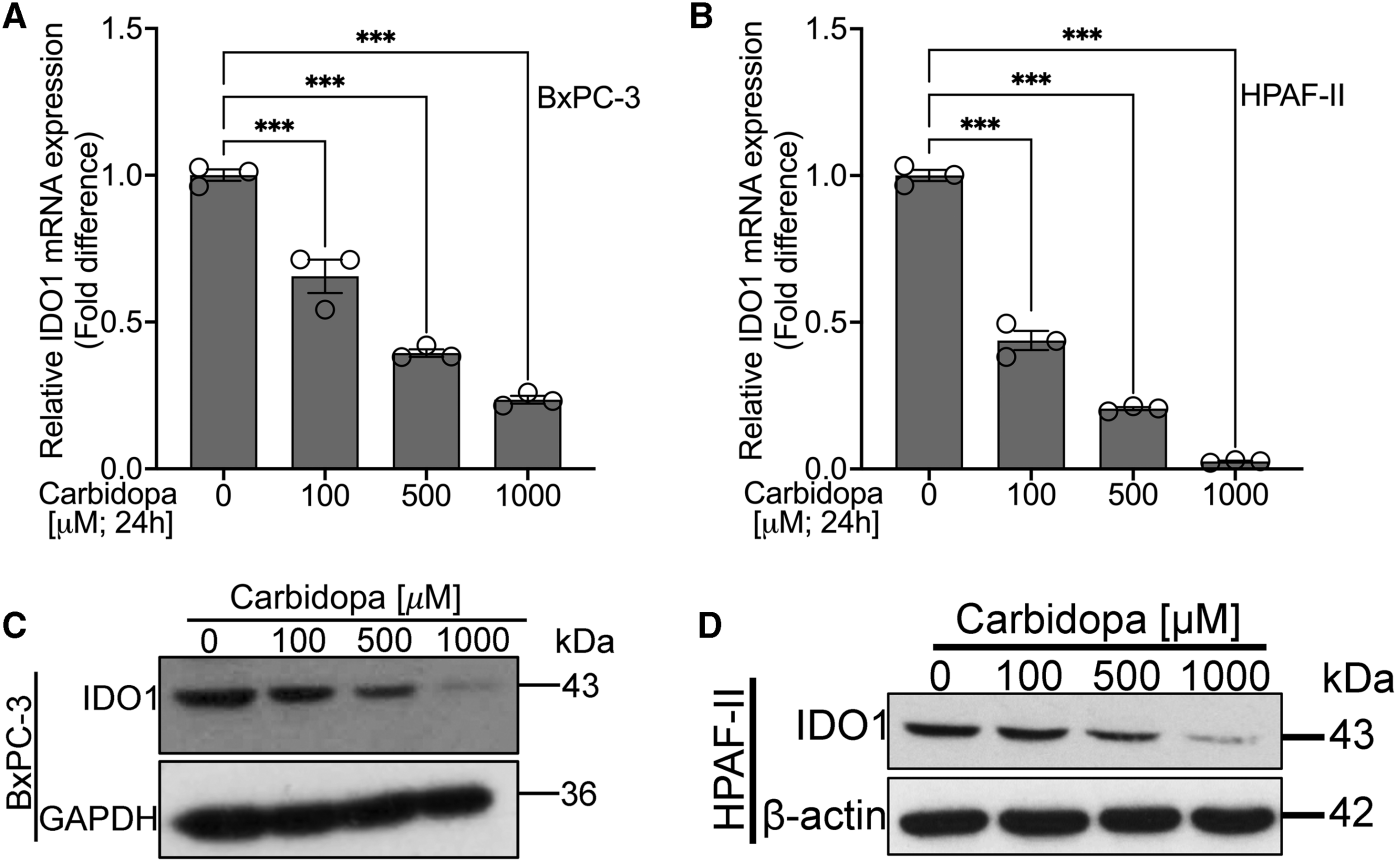
Carbidopa decreases IDO1 mRNA and IDO1 protein. (**A** and **B**) Real-time PCR showing relative IDO1 mRNA levels in BxPC-3 and HPAF-II cell lines following 24 h treatment with 100 µM, 500 µM and 1000 µM of carbidopa. (**C** and **D**) Western blotting showing IDO1 protein levels in BxPC-3 and HPAF-II cell lines following 24 h treatment with 100 µM, 500 µM and 1000 µM of carbidopa. β-actin was used as an endogenous control. For all *in vitro* studies *n* = 3. Data are means ± SEM. Statistical significance for (**A**) and (**B**) were assessed using one-way ANOVA with Dunnett's *post hoc* test (*** P < 0.001).

We have already identified carbidopa, an FDA-approved drug for Parkinson's disease, to have an anticancer effect in pancreatic cancer via its role as an AhR agonist [29]. Additionally, based on the computer modeling and the enzymatic assay with a recombinant IDO1, we do know that carbidopa has a direct inhibitory effect on IDO1 activity. In the present study, we asked the question if carbidopa has any effect on IDO1 expression because of its role as an agonist for AhR, a transcription factor. To address this question, we selected IDO1-positive BxPC-3 and HPAF-II cells and treated them with various concentrations of carbidopa (100 µM, 500 µM and 1000 µM) for 24 h. Post-treatment, total RNA was isolated and Real-Time RT-qPCR was conducted to assess the effect of carbidopa on IDO1 mRNA. We found that carbidopa treatment led to a significant dose-dependent reduction in IDO1 mRNA in both BxPC-3 and HPAF-II cell lines (Figure 2A,B). To determine if the observed decrease in IDO1 mRNA levels resulted in corresponding changes in IDO1 protein levels, we performed Western blotting using the protein lysates from control and treated cells. We found a decrease in IDO1 protein as well in carbidopa-treated cells (Figure 2C,D). These studies show that carbidopa not only interferes with IDO1 enzymatic activity by direct interaction but also suppresses IDO1 expression at the mRNA and protein levels.

### Carbidopa suppresses IFN-γ-induced IDO1 in PDAC cell lines

**Figure 3.**
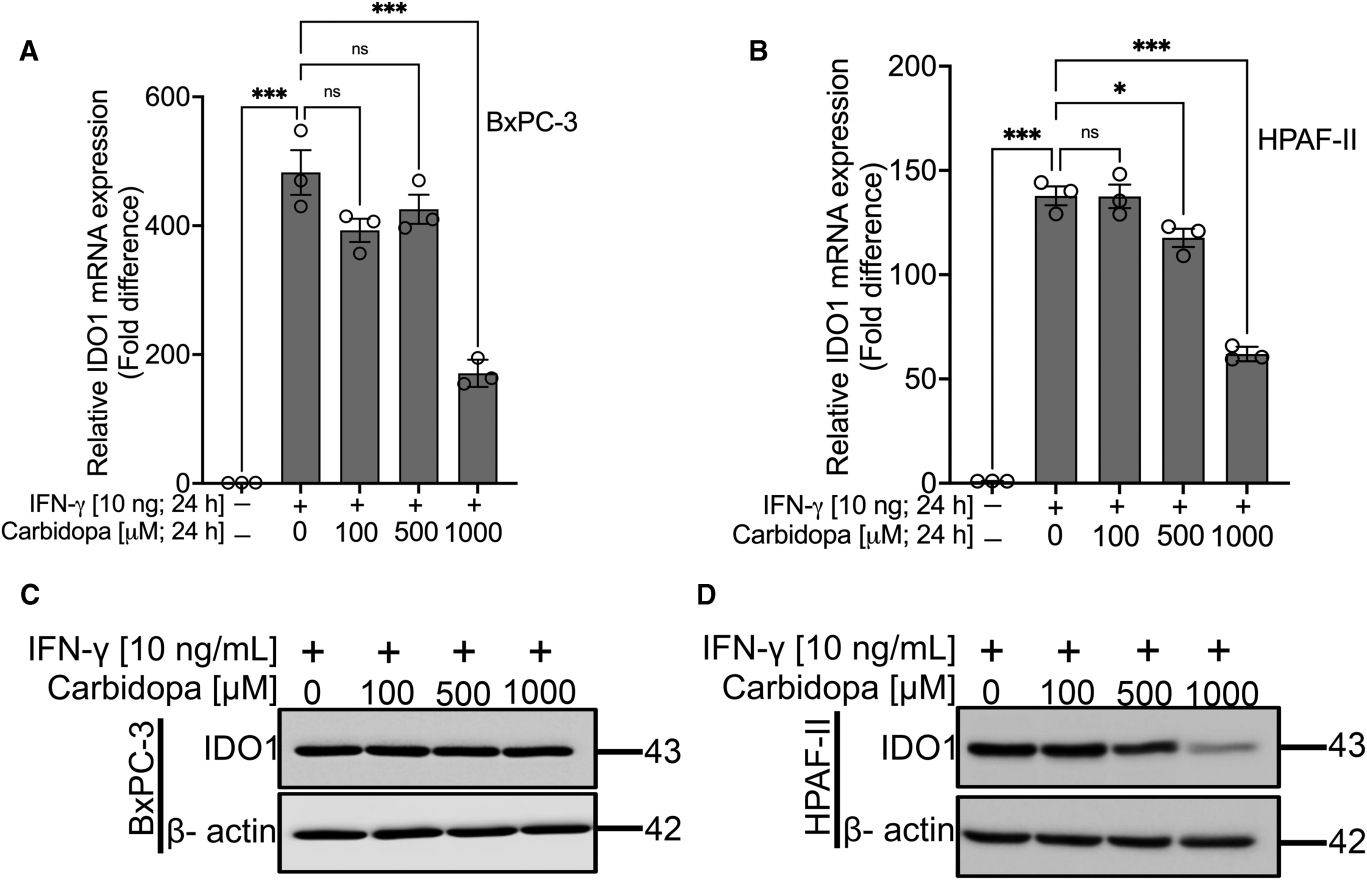
Carbidopa suppresses IFN-γ-induced IDO-1. (**A** and **B**) Real-Time PCR showing relative IDO1 mRNA levels in BxPC-3 and HPAF-II cell lines cultured either in the absence or presence of IFN-γ and various concentrations of carbidopa (100 µM, 500 µM and 1000 µM) for 24 h. (**C** and **D**) Western blotting showing IDO1 protein levels in BxPC-3 and HPAF-II cell lines cultured either in the absence or presence of IFN-γ and various concentrations of carbidopa (100 µM, 500 µM and 1000 µM) for 24 h. β-actin was used as an endogenous control. For all *in vitro* studies *n* = 3. Data are means ± SEM. Statistical significance for (**A**) and (**B**) were assessed using one-way ANOVA with Tukey's *post hoc* test (*P < 0.05; *** *P* < 0.001; ns > 0.05). ns; not significant.

Since the data presented above show results with constitutively expressed IDO1 in BxPC-3 and HPAF-II cells, we were curious to find out whether carbidopa is capable of inhibiting cytokine-inducible IDO1 as well. IFN-γ is a potent inducer of IDO1. Therefore, BxPC-3 and HPAF-II cells were treated with 10 ng/ml of IFN-γ in the absence or in the presence of varying concentrations of carbidopa (100 µM, 500 µM and 1000 µM) for 24 h. As expected, IFN-γ treatment led to a dramatic enhancement in IDO1 mRNA (>400-fold in BxPC-3 and >100-fold in HPAF-II cells). Real-time RT-qPCR data showed the ability of carbidopa to decrease IDO1 mRNA even in the presence of IFN-γ (Figure 3A,B). However, carbidopa did not decrease the IDO1 protein in IFN-γ-exposed BxPC-3 cells (Figure 3C) but it did in HPAF-II cells (Figure 3D). This could be due to the difference in the relative expression of IDO1 mRNA levels between the two cell lines in the presence of IFN-γ but in the absence of carbidopa. The induction of IDO1 mRNA by IFN-γ was ∼450-fold in BxPC-3 cells whereas the corresponding value was <150-fold in HPAF-II cells. The relatively higher basal levels of mRNA in BxPC-3 cells than in HPAF-II cells could be at least one of the reasons for the lack of any noticeable effect of carbidopa on IDO1 protein levels in the former cell line.

### Carbidopa suppresses both constitutive and IFN-γ-induced IDO1 expression

**Figure 4.**
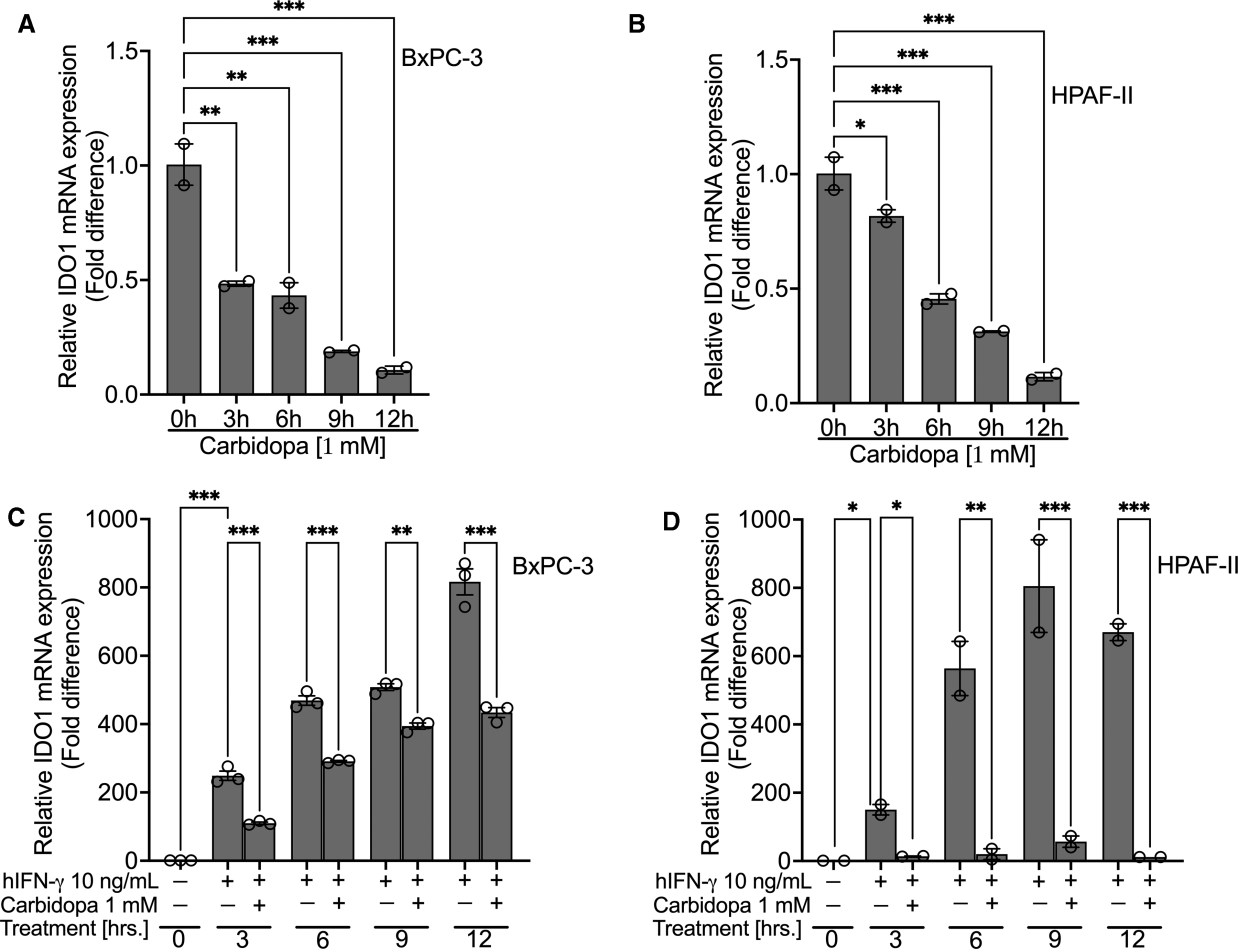
Carbidopa suppresses both constitutive as well as IFN-γ-induced IDO-1. (**A** and **B**) Real-Time PCR showing time-dependent suppression of constitutive IDO1 in BxPC-3 and HPAF-II cell lines following treatment with 1 mM of carbidopa. (**C** and **D**) Real-Time PCR showing time-dependent inhibition of IFN-γ-induced IDO1 in BxPC-3 and HPAF-II cell lines following treatment with 1 mM of carbidopa. For all *in vitro* studies *n* = 3. Data are means ± SEM. Statistical significance were assessed using one-way ANOVA with Dunnett's *post hoc* test for (**A**) and (**B**) and Tukey's *post hoc* test for (**C**) and (**D**) (*P < 0.05; ** *P* < 0.01; *** *P* < 0.001).

Next, we investigated the time course of carbidopa effect on IDO1 expression, both in the absence and presence of IFN-γ. BxPC-3 and HPAF-II cell lines, both expressing IDO1 constitutively, were treated with carbidopa (1 mM) for increasing time periods (0, 3, 6, 9 and 12 h). Following the treatment, we monitored IDO1 mRNA by RT-qPCR. Carbidopa remarkably down-regulated IDO1 mRNA as early as 3 h treatment, and the effect increased further with longer periods of treatment (Figure 4A,B). Upon IFN-γ treatment, IDO1 was induced in both cell lines as expected. Carbidopa treatment caused a significant reduction in IDO1 mRNA at all time points in both cell lines, however, it was not time-dependent (Figure 4C,D).

### Carbidopa attenuates tumor growth in athymic nude mice

**Figure 5.**
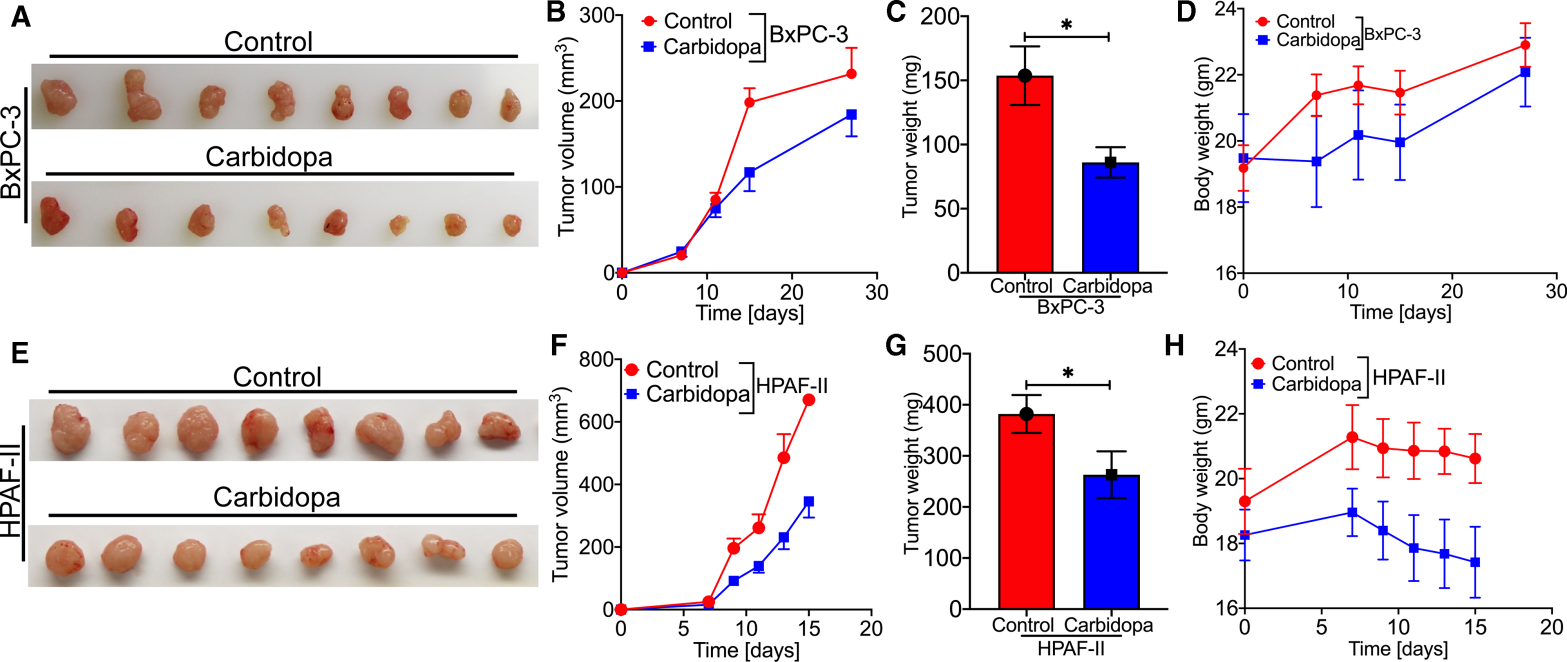
Carbidopa-mediated IDO1 suppression attenuates tumor growth in athymic nude mice. BxPC-3 and HPAF-II cells were subcutaneously implanted in athymic nude mice. Representative photographs of harvested tumors from control and carbidopa-treated mice bearing BxPC-3 cells (**A**) and HPAF-II cells (**E**). Tumor growth curves of BxPC-3 (**B**) and HPAF-II (**F**) tumor-bearing nude mice treated with regular drinking water or carbidopa. Tumor weights between control and carbidopa-treated mice bearing BxPC-3 (**C**) and HPAF-II cells (**G**). Evaluation of mouse body weights during the xenograft experiments: BxPC-3 (**D**); HPAF-II (**H**). Data are given as mean ± SEM. * P < 0.05.

The studies described thus far show that carbidopa suppresses the expression of IDO1 in PDAC cells. To determine if this effect has any relevance to tumor growth in PDAC, we chose to perform a subcutaneous xenograft implantation of human PDAC cells in athymic nude mice as the *in vivo* model for PDAC. Female athymic nude mice were injected with IDO1-positive BxPC-3 and HPAF-II cells and then randomly divided into control and treatment groups a week after the cancer cell injection. The mice in the control groups received regular drinking water while the treatment groups received 1 mg/ml of carbidopa in drinking water. As expected, carbidopa treatment led to a significant reduction in tumor growth as evident from changes in tumor volume and tumor weight in cases of both BxPC-3 (Figure 5A–C) and HPAF-II cells (Figure 5E–G). There was no significant change in body weight of the mice with and without carbidopa treatment (Figure 5D,H) in both cell lines, suggesting no obvious toxicity of Carbidopa. To simulate carbidopa administration in human patients, subsequent subcutaneous xenograft experiment was conducted in athymic nude mice wherein carbidopa was administered to the mice through oral gavage @ 1 mg in 200 µl of canola oil suspension once a day. Control group was administered with 200 µl of canola oil through oral gavage. Similar results were obtained in terms of tumor growth and body weight in both BxPC-3 (Supplemental Figure S1A–C) and HPAF-II cells (Supplemental Figure S1E–G).

### IDO1 expression in control and carbidopa-treated tumors

**Figure 6.**
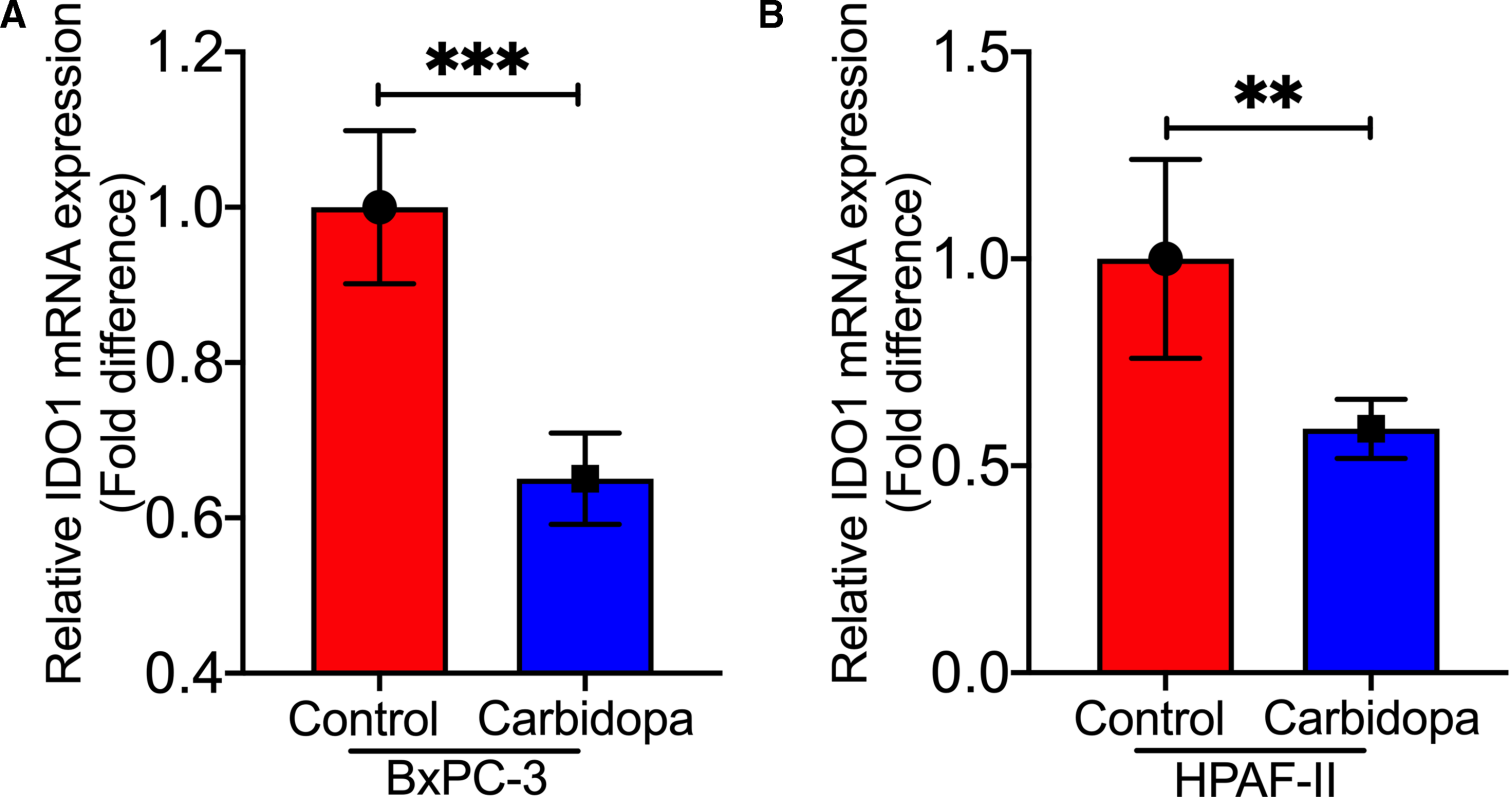
Carbidopa suppressed IDO1 expression in tumor xenograft samples. (**A** and **B**) Real-Time PCR showing relative IDO1 mRNA expression in control and carbidopa-treated BxPC-3 and HPAF-II tumor xenograft samples extracted from the athymic nude mice. Data are given as mean ± SEM. ** P < 0.01, *** *P* < 0.001.

To determine if the observed inhibition in tumor growth by carbidopa is associated with changes in IDO1 expression, we performed RT-qPCR using tumor samples from control and carbidopa-treated mice from both the experiments, i.e. carbidopa in drinking water and carbidopa through oral gavage. Since the tumors arose from human PDAC cells in a mouse xenograft, we monitored the expression of IDO1 mRNA specifically in tumor cells by using primers specific for human IDO1. It was interesting to observe that in both the experiments carbidopa treatment led to a significant decrease in the levels of human IDO1 mRNA when compared with control tumors (Figure 6A,B and Supplemental Figure S1D,H). These data suggest that carbidopa-mediated suppression of IDO1 expression in cancer cells might contribute to the decreased tumor growth observed in carbidopa-treated xenografted mice.

### Effect of carbidopa on xenografted PDAC growth with BxPC-3 cells in the absence and presence of gemcitabine

We next sought to investigate whether carbidopa would work in synergy with gemcitabine, a standard chemotherapeutic agent for PDAC, to exhibit a more pronounced anticancer effect. For this, we performed a subcutaneous xenograft in athymic nude mice using BxPC-3 as the model cell line. BxPC-3, apart from being IDO1-positive cell line, is also not as aggressive as HPAF-II in tumor formation and growth in xenografts; this negates the need for early termination of the experiment and potential failure to detect any synergistic effect. This was the basis for choosing BxPC-3 as the model cell line. A week after the tumor cell injection, the mice were divided into four groups: (i) control, (ii) carbidopa, (iii) gemcitabine, and (iv) carbidopa + gemcitabine. The treatment regimen consisted of regular drinking water for the control group, 1 mg/ml of carbidopa in drinking water for the carbidopa group, 50 mg/kg of gemcitabine i/p once a week for the gemcitabine group, and a combination of carbidopa (1 mg/ml in drinking water) plus gemcitabine (50 mg/kg i/p once a week) for the combination group. As expected, carbidopa and gemcitabine individually led to a significant inhibition in tumor growth as shown by changes in tumor volume and tumor weight (Supplemental Figure S2A,B). However, to our surprise, the combination therapy was not superior to either of the monotherapies. While this shows that carbidopa does not synergize with gemcitabine, it is evident that carbidopa by itself is as good as gemcitabine in attenuating tumor growth.

### Carbidopa-mediated inhibition of IDO1 in the tumor cells inhibits mTORC1 signaling pathway

**Figure 7.**
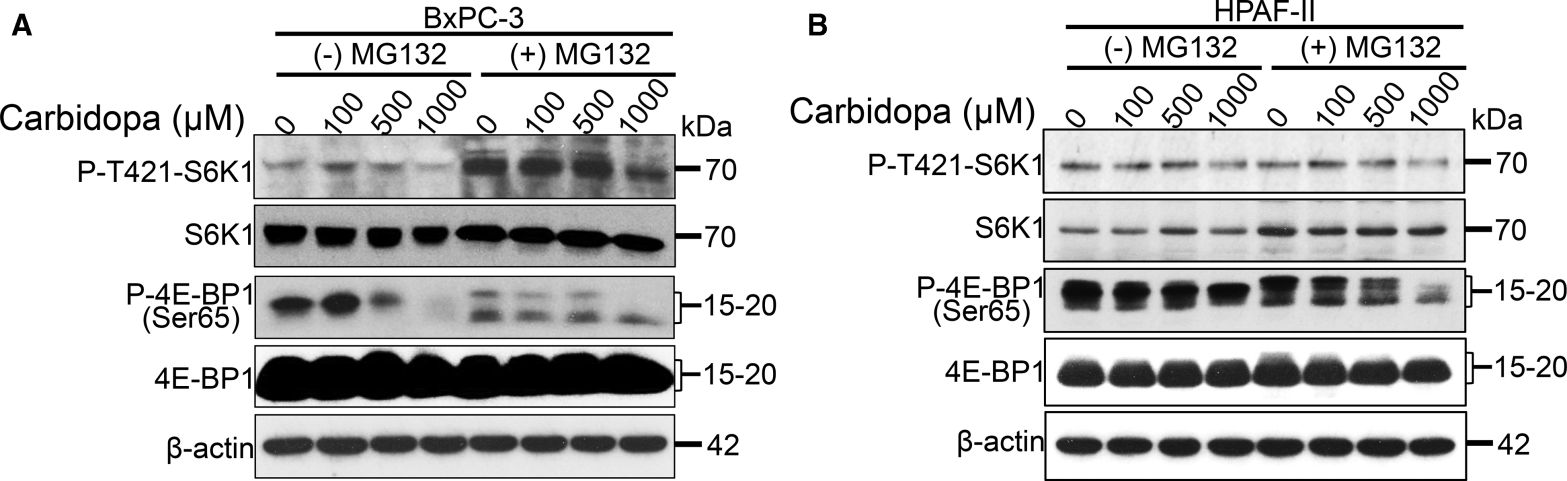
Carbidopa-mediated suppression of IDO1 in tumor cells inhibits mTORC1 signaling pathway. (**A** and **B**) Western blotting showing protein expression of S6K and 4EBP1 (total and phospho-form) in carbidopa-treated BxPC-3 and HPAF-II cell lines, which were also treated with MG132 to rule out protein degradation. β-actin was used as an endogenous control.

Literature evidence has shown tryptophan depletion due to overactivation of IDO1 to inhibit mTORC1 signaling pathway [22]. This is due to activation of the integrated stress response kinase GCN2 [30,31], which phosphorylates and inhibits the eukaryotic initiation factor 2α (eIF2α), blocking protein synthesis and arresting cell growth [32]. This is critical from an immune cell perspective especially for the T-cells that are highly dependent on tryptophan for their growth and proliferation. If this is true in our experimental system, decreased expression of IDO1 in carbidopa-treated PDAC cells would reverse this process resulting in stimulation of mTORC1 signaling, which would be favorable for tumor growth, an effect opposite of what we observed. mTORC1 is a master regulator of protein synthesis wherein its signaling is regulated by several factors including, but not limited to, amino acids, nutrients, and growth factors [2,33,34]. Since we observed an inhibitory effect of carbidopa on tumor growth, we wanted to monitor the mTORC1 signaling pathway in control and carbidopa-treated PDAC cells. For that, BxPC-3 and HPAF-II cells were cultured with and without various concentrations of carbidopa for 24 h. In another set of experiment, MG132, a ubiquitin proteasome inhibitor was added in the last 6 h of carbidopa treatment to ensure that the target proteins were not degraded following carbidopa treatment. As a read-out for activation of mTORC1 pathway, we checked the phosphorylation status of p70 ribosomal S6 kinase (S6K) and 4E-binding protein 1 (4EBP1). Increased phosphorylation of these downstream effectors of mTORC1 would indicate activation of the pathway. We found that carbidopa treatment reduced the phosphorylation status of both S6K and 4EBP1 in both the cell lines. There was no obvious change in the total protein levels, which was further supported by the MG132 result. Taken together the result indicate that carbidopa treatment inhibits mTORC1 signaling pathway (Figure 7A,B).

### Involvement of AhR in carbidopa-mediated suppression of IDO1 expression in PDAC cells

**Figure 8.**
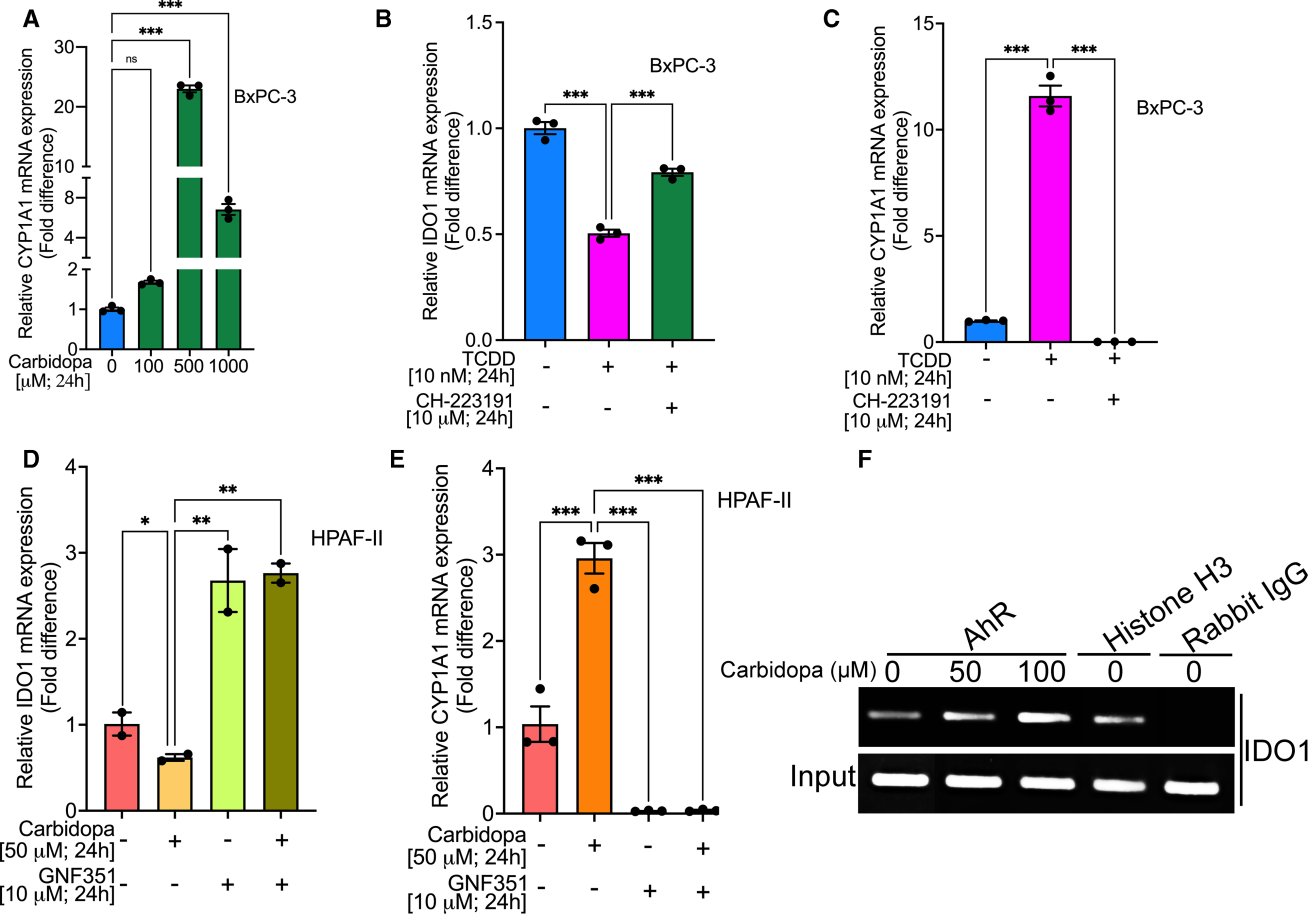
AhR mediates carbidopa-mediated IDO1 suppression in PDAC cells. (**A**) Real-time PCR showing relative mRNA expression of CYP1A1 in BxPC-3 cells treated with various concentrations of carbidopa (100 µM, 500 µM and 1000 µM) for 24 h. (**B** and **C**) Real-time PCR showing relative mRNA expression of IDO1 and CYP1A1 in BxPC-3 cells cultured with 10 nM TCDD, either in the presence or absence of an AhR antagonist, CH-223191. (**D** and **E**) Real-time PCR showing relative mRNA expression of IDO1 and CYP1A1 in HPAF-II cells cultured with 50 µM carbidopa, either in the presence or absence of an AhR antagonist, GNF351. (**F**) ChIP assay showing AhR binding to the *IDO1* promoter. For all *in vitro* studies *n* = 3. Data are means ± SEM. Statistical significance were assessed using one-way ANOVA with Dunnett's *post hoc* test for (**A**) and Tukey's *post hoc* test for (**B**–**E**) (* P < 0.05; ** P < 0.01; *** *P* < 0.001; ns > 0.05). ns; not significant.

We have already shown that carbidopa is an AhR agonist and that it inhibits pancreatic cancer growth [29]. In the present study, we find that carbidopa interferes with IDO1 expression in PDAC cells, but we do not know if activation of AhR has any role in this phenomenon. It is possible that carbidopa elicits its anticancer activity at least partly by AhR-mediated down-regulation of IDO1 expression. To evaluate this possibility, we first checked the expression of CYP1A1, a known AhR target gene [35] in control and carbidopa-treated PDAC cells. These studies showed that CYP1A1 mRNA levels were markedly higher in carbidopa-treated cells than in control cells (Figure 8A), clearly indicating activation of AhR. To determine if the carbidopa-mediated suppression of IDO1 and activation of AhR are merely correlative or actually represent a cause-effect relationship, we studied the effect of TCDD, a bone-fide and widely used potent AhR agonist, on IDO1 expression in BxPC-3 cells. We found that TCDD treatment did suppress the expression of IDO1 in these cells (Figure 8B). To further confirm the involvement of AhR in this process, we examined the ability of CH-223191, an AhR antagonist, on TCDD-mediated suppression of IDO1 expression. Blockade of AhR activity with this antagonist significantly reversed the effect of TCDD on IDO1 expression (Figure 8B). This was further corroborated by showing the ability of this antagonist to reverse the TCDD-mediated induction of the AhR target CYP1A1 (Figure 8C). We further wanted to validate these findings in HPAF-II cells, another PDAC cell line. HPAF-II cells were treated with carbidopa in the presence or absence of GNF351, another highly potent AhR antagonist. As in the previous experiments, carbidopa decreased IDO1 mRNA (Figure 8D) and increased CYP1A1 mRNA (Figure 8E). GNF351 reversed both of these effects (Figure 8D,E). Treatment with GNF351 alone induced IDO1 mRNA and suppressed CYP1A1 mRNA, providing evidence for constitutively active AhR and its differential effects on IDO1 and CYP1A1 expression. We also performed chromatin immunoprecipitation (ChIP) assay to show the direct interaction of AhR with the promoter of IDO following carbidopa treatment. AhR is a transcription factor and, upon activation by its ligand, gets translocated to the nucleus wherein it binds to the xenobiotic (dioxin)-responsive element in its target genes and either induces or suppresses their expression. The interaction of AhR with IDO1 upon activation by its ligands, either endogenous or exogenous and the transcriptional regulation of IDO1 is already known [36,37]. However, what is not known is whether carbidopa-mediated activation of AhR leads to its interaction with IDO1 or not. The ChIP assay clearly showed that compared with the control, carbidopa treatment clearly enhanced AhR interaction with IDO1 promoter (Figure 8E) suggesting that the carbidopa-mediated IDO1 inhibition is AhR regulated. Kynurenine, a tryptophan metabolite, and a known endogenous ligand for AhR is known to induce interaction of AhR and IDO and thereby maintain the IDO1 expression status in cells [36]. Since culture media contains several amino acids including Trp, IDO1 can catabolize Trp leading to production of kynurenine, which in turn can lead to AhR/IDO1 interaction, thereby maintaining the basal expression of IDO1. Taken together, these data show that carbidopa-mediated suppression of IDO1 expression is AhR regulated.

### Role of JAK1/STAT1 pathway in carbidopa-mediated suppression of IDO1 expression

**Figure 9.**
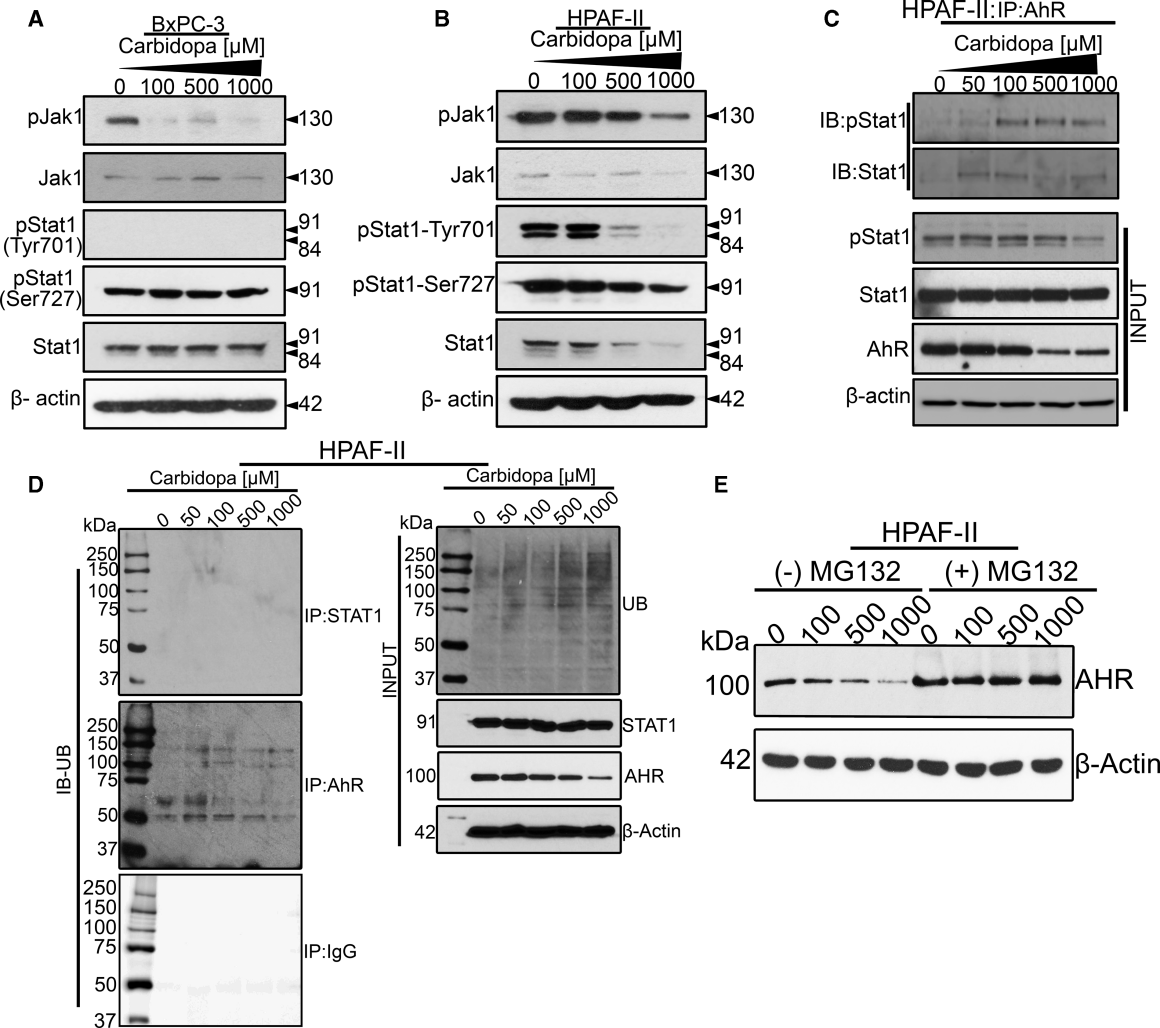
Carbidopa-mediated IDO1 suppression is also mediated by AhR indirectly by interfering with the JAK1/STAT1 signaling pathway. (**A** and **B**) Western blotting showing protein expression of JAK1, STAT1 (both phospho-form and total) in BxPC-3 and HPAF-II cells lines treated with various concentrations of carbidopa (100 µM, 500 µM and 1000 µM) for 24 h. β-actin was used as an endogenous control. (**C**) Immunoprecipitation data showing carbidopa-mediated promotion of AhR and STAT1 interaction in HPAF-II cells. (**D**) Immunoprecipitation data showing ubiquitinated STAT1 and ubiquitinated AhR in HPAF-II cells treated with various concentrations of carbidopa. (**E**) Western blotting showing AhR expression following carbidopa treatment, either in the presence or absence of MG132.

The induction of IDO1 is regulated mainly by both IFN-γ-dependent and -independent mechanisms [38–41]. The binding of IFN-γ to its cell-surface receptor leads to the activation of JAK1 by autophosphorylation and subsequent phosphorylation of downstream substrates such as STAT1, which results in the activation of the transcription factor IRF-1 [42]. IRF-1 can bind ISRE sequences of IDO1 promoter to induce IDO1 transcription. This led us to hypothesize that carbidopa could possibly regulate IDO1 expression in PDAC cells by negatively modulating this JAK1/STAT1 signaling pathway. Cancer cells are known to secrete cytokines, including interferons, which in an autocrine manner can regulate the constitutive IDO1 expression via the JAK1/STAT1 signaling pathway. There is also evidence that carbidopa inhibits the expression and secretion of IFN-γ and IL-6 [43], which are known inducers of IDO1 expression. Therefore, we monitored the phosphorylation status of JAK1, JAK2 and STAT1 in control and carbidopa-treated BxPC-3 and HPAF-II cells. These studies showed that carbidopa treatment decreased the phosphorylation of JAK1 in both cell lines (Figure 9A,B). Neither non-phosphorylated nor phosphorylated form of JAK2 was detectable in these cells. Phospho-STAT1 was decreased, both at tyrosine 701 and at serine 727, in HPAF-II cells upon treatment with carbidopa. However, in BxPC-3 cells, phospho-STAT1 at tyrosine 701 was not detectable and phospho-STAT1 at serine 727 remained unchanged in carbidopa-treated cells. Total JAK1 and STAT1 proteins remained unchanged in response to carbidopa treatment. These data clearly indicated that carbidopa inhibited the JAK1/STAT1 signaling pathway.

Our next question was whether the inhibition of JAK1/STAT1 pathway by carbidopa was AhR-mediated. AhR is known to have dual functions in controlling intracellular protein levels, serving both as a transcription factor and as a ligand-dependent E3 ubiquitin ligase. Because AhR can function as a ubiquitin ligase [44], it can act on JAK1 and STAT1 proteins to promote their proteasome-mediated degradation. Therefore, it is possible that the observed decrease in JAK1 and STAT1 proteins following carbidopa treatment could be a result of direct interaction of AhR leading to their degradation. In support of our hypothesis, literature evidence already shows that AhR forms a complex with STAT1 [45,46]. To test this, HPAF-II cells were treated with increasing concentrations of carbidopa, followed by immunoprecipitation and Western blotting to monitor the interaction between AhR and STAT1. The results demonstrated that carbidopa treatment enhanced the interaction of AhR with STAT1, both phospho as well as the total form (Figure 9C). However, AhR did not interact with JAK1 or JAK2 (data not shown). To further characterize if this interaction leads to ubiquitination and degradation of STAT1, immunoprecipitation was performed in HPAF-II cells treated with and without carbidopa using anti-STAT1 antibody followed by Western blotting to check for ubiquitin. Interestingly, the data showed that STAT1 was not ubiquitinated following carbidopa treatment and therefore not degraded by the ubiquitin-ligase activity of AhR (Figure 9D). However, it's possible that AhR/STAT1 interaction interferes with the JAK/STAT signaling cascade, ultimately affecting IDO1 transcription. Furthermore, since the AhR level in the input sample was decreased by carbidopa treatment, we were curious to investigate if AhR itself gets ubiquitinated and degraded after binding with carbidopa. Our immunoprecipitation data and the Western blotting data with and without MG132 clearly indicate that AhR protein eventually gets ubiquitinated and degraded following carbidopa treatment (Figure 9D). This suggests that carbidopa-mediated activation of AhR follows the classic adaptive AhR-mediated signaling wherein the hallmarks include AhR activation by its ligand, nuclear accumulation and dimerization to ARNT, binding of the AhR-ARNT dimer to xenobiotic response elements, regulation of gene expression, and ubiquitin-mediated degradation of AhR [47].

Taken together, these studies show that AhR is involved in carbidopa-mediated IDO1 suppression by at least two mechanisms, one involving the direct action of AhR as a transcription factor and the other involving the AhR/STAT1 interaction that could affect the JAK1/STAT1 signaling cascades affecting IDO1 transcriptional activation.

## Discussion

IDO1 plays a role both in a physiological as well as in a pathological setting. Discovered in the 1950s, IDO1 is the most fully characterized enzyme in the kynurenine biosynthesis pathway. From a pathological standpoint, specifically from a cancer perspective, IDO1 is known to be significantly up-regulated in various cancer types like the ovarian cancer, malignant melanoma, pediatric and adult acute myelogenous leukemia, colorectal cancer, prostate cancer, endometrial cancer as well as in pancreatic cancer and is known to affect the overall patient survival [7–17]. Though IDO1 is also overexpressed by the tumor cells, what has caught the most attention is its overexpression in the DCs that depletes tryptophan in the TME leading to T-cell anergy and immune tolerance by the tumor cells. It is not a wonder therefore that immunotherapy targeting IDO1 has been the primary focus for the last several years. However, several recent reports have shown the therapeutic benefit of targeting IDO1 in cancer cells as well. In fact, a recent study has revealed that the tumor cell associated IDO1 has a unique functionality in terms of tryptophan metabolism to provide one carbon source for purine nucleotide synthesis to sustain their proliferation. This suggests that tumor cell associated IDO1 also has a tumor-promoting effect via a non-immune related function and therefore, apart from targeting IDO1 in the DCs, targeting tumor cell IDO1 might prove as a beneficial therapeutic strategy as well.

PDAC is a therapeutic challenge with a survival rate only in single digits [48]. Immunotherapy targeting IDO1 has potential in cancer treatment, with some IDO1 inhibitors have gone beyond phase 2 clinical trials. But PDAC is unique from the rest of the cancers characterized by hypovascularity, rich stromal elements and low immunogenicity and therefore the immunotherapy results have been disappointing. Since PDAC cells also overexpress IDO1, and because IDO1 has a context-dependent function, would it rather be more beneficial to inhibit IDO1 in tumor cells? Of course, from a clinical standpoint, it will be hard to pin-point whether the observed anticancer effect is due to IDO1 inhibition in the immune cells or in the tumor cells. Nevertheless, experimental evidence supporting the rationale for using IDO1 as a drug target for cancer therapy would be welcome irrespective of whether it is the immune-cell IDO1 or the cancer-cell IDO1 that elicits the therapeutic effect. Here we explored the potential of the FDA-approved drug carbidopa in pancreatic cancer therapy as a blocker of IDO1 expression and function.

The findings of the present study can be summarized as follows: (a) IDO1 is up-regulated in PDAC cell lines and primary tumors from PDAC patients and the expression exhibits a reciprocal correlation with patient survival; (b) Carbidopa inhibits both constitutive and IFN-γ-induced IDO1 expression in a dose- and time- dependent manner; (c) Carbidopa inhibits tumor growth in a xenograft mouse model and the observed anticancer effect is at least partly IDO1-mediated; (d) despite its inability to synergize with gemcitabine, carbidopa by itself attenuates tumor growth; (e) Carbidopa treatment did not stimulate, rather inhibited, mTORC1 signaling pathway; and (f) AhR regulates carbidopa-mediated suppression of IDO1 expression in PDAC cells, directly by its function as a transcription factor and indirectly by the carbidopa-mediated interaction of STAT1 with AhR that could affect the JAK1/STAT1 signaling cascades affecting IDO1 transcriptional activation.

The anticancer role of carbidopa has already been documented. We have shown that carbidopa as an AhR agonist attenuates pancreatic cancer growth [29] while other investigators have shown that carbidopa suppresses prostate cancer growth via AhR-mediated ubiquitination and degradation of androgen receptor [49]. In the current study, we have unveiled another mechanism for the anticancer efficacy of carbidopa, namely suppression of IDO1. With these many attributes, carbidopa is surely a potential candidate for cancer therapy. Though carbidopa appears to have multiple targets, AhR seems to be important for its anticancer effect. AhR is a transcription factor highly expressed in various cancer types including PDAC. It is normally located in the cytoplasm when unliganded but upon binding to its agonist, it gets translocated to the nucleus and regulates target gene expression. In the cytoplasm, AhR functions as a component of E3 ubiquitin ligase. Our studies show that IDO1 is one of the downstream targets for the nuclear functions of AhR. Kynurenine is the product of IDO1 action on tryptophan. The AhR-kynurenine axis serves as a feedback loop for the induction of IDO1 [50,51]. Therefore, given this functional circuitry between AhR and IDO1 and their functional relevance to cancer, our studies provide a novel insight into the anticancer efficacy of carbidopa.

Literature evidence has implicated tryptophan depletion due to overactivation of IDO1 to inhibit mTORC1 signaling pathway. This is critical from an immune cell perspective, especially for the T-cells that are highly dependent on tryptophan for their growth and proliferation. However, according to this rationale, inhibition of IDO1 would reverse this process resulting in stimulation of mTORC1 signaling pathway, which on the contrary would be pro-tumorigenic [52,53]. But, our studies show that carbidopa in fact suppresses mTORC1 signaling. We speculate that apart from suppressing IDO1 expression and function, carbidopa serve to inhibit tryptophan entry into tumor cells and tumor-associated immune cells by blocking either the Tryptophan-selective transporter [54] or other amino acid transporters such as SLC6A14 that transport Tryptophan. Our previous study has shown that IFN-γ induces the tryptophan-selective transporter in the DCs [54]. Though the molecular identity of the transporter is not known yet, we believe that it should be present in any cells that express IDO1 as an entry mechanism for tryptophan. SLC6A14 is a broad-spectrum amino acid transporter that is significantly up-regulated in PDAC as well as many cancer types [55–59]. It transports all amino acids except aspartate and glutamate. If carbidopa were to inhibit these transporters, cellular concentrations of tryptophan will decrease, which will suppress mTORC1 pathway. Though leucine and arginine are better known for their ability to activate mTORC1 signaling, tryptophan also has been implicated in the regulation of this signaling pathway [60,61].

The JAK1/STAT1 pathway is the canonical cytokine-induced signaling that is constitutively active in cancer cells. This pathway is also known to regulate IDO1 expression upon binding of IFN-γ to its cognate receptor, which induces signaling cascades leading to sequential activation and phosphorylation of JAKs and STATs, ultimately leading to IDO1 transcriptional activation. That said, interferon-independent mechanisms such as LPS-stimulated p38 mitogen-activated protein kinase, phosphatidylinositol 3-kinase, and nuclear factor-kB (NF-kB) pathways are also known to induce IDO1. Here we show that carbidopa suppresses IDO1 via AhR activation, with at least two different downstream effects. AhR forming a complex with STAT1 is already known, but here we show that carbidopa promotes this interaction.

It has to be pointed out that even though carbidopa was used at high micromolar concentrations for *in vitro* studies in the present study, the dose shown to be effective as an anticancer agent in our *in vivo* mouse studies is therapeutically relevant to humans. Carbidopa is used in patients with Parkinson's disease at a maximum dose of 200 mg/day; however, studies have shown that it can be used as high as 450 mg/day without any noticeable side effect [62]. This dose translates to 1.5 mg/mouse (body weight, ∼20 g) with corrections for difference in body surface area between mice and humans. This is similar to the dose used in our *in vivo* studies in mice for evaluation of carbidopa's efficacy for PDAC (1 mg/ml in drinking water; normal intake of water is ∼3 ml/day/mouse & 1 mg total dose via oral gavage). Therefore, the dose of carbidopa that shows significant anticancer effect is achievable for patients with pancreatic cancer, which makes the findings of the present study clinically significant.

## Conclusion

In summary, we find that carbidopa-mediated IDO1 suppression attenuates PDAC growth. Mechanistically, carbidopa activates AhR, which in turn down-regulates IDO1 expression, directly as a transcription factor and indirectly by inhibiting the JAK1/STAT1 pathway. We have already published reports on the anticancer efficacy of carbidopa to treat PDAC in mouse models; the present study provides a mechanistic insight into the anticancer actions of drug, underscoring the repurposing potential for this anti-Parkinsonian drug for cancer treatment.

## Materials and methods

### Materials

Carbidopa was purchased from 3B Scientific Corporation Limited (Wuhan, China). Bicinchoninic acid protein assay reagent was from Pierce Chemicals (Rockford, IL, U.S.A.). Primary antibodies like β-actin mouse monoclonal (#sc-47778) was from Santa Cruz Biotechnology (Dallas, Texas, U.S.A.) while IDO1 mouse monoclonal (#86630S), Jak1 rabbit polyclonal (#3332S), Phospho-Jak1 rabbit polyclonal (#74129S), Stat1 rabbit polyclonal (#9172S), Phospho-Stat1 (Tyr701) rabbit monoclonal (#7649S), Phospho-Stat1 (Ser727) rabbit monoclonal (#8826S), phospho-p70 S6 kinase (Thr421/Ser424) (#9204), p70 S6 kinase (E8K6T) XP rabbit monoclonal (#34475), phospho-4E-BP1 (Ser65) (#9451), 4E-BP1 (53H11) rabbit monoclonal (#9644), AhR (D5S6H) rabbit monoclonal (#83200), ubiquitin (P4D1) mouse monoclonal (#3936) antibodies were procured from Cell Signaling Technology (Danvers, MA, U.S.A.). Secondary antibodies, goat anti-rabbit IgG (#170-6515) and goat anti-mouse IgG (#170-6516), were procured from Bio-Rad Laboratories (Hercules, CA, U.S.A.).

### Cell culture

HPDE, a human normal pancreatic ductal epithelial cell line, was kindly provided by Dr. Ming Tsao, Ontario Cancer Institute (Toronto, Canada). hTERT-HPNE, another human normal pancreatic ductal epithelial cell line, as well as human pancreatic cancer cell lines AsPC-1, BxPC-3, Capan-1, Capan-2, CFPAC-1, HPAF-II, MIA PaCa-2, PANC-1, Panc 10.05, and SU.86.86 were obtained from ATCC. These cell lines were used within 10–20 passages. The ATCC has done morphological, cytogenetic and DNA profile analyses for characterization of these cell lines. HPDE cells were cultured in Keratinocyte Serum Free Media supplemented with epidermal growth factor and bovine pituitary extract. hTERT-HPNE cells were maintained in 75% Dulbecco's Modified Eagle's Medium (DMEM) without glucose plus 25% Medium M3 Base with the following additives: 5% FBS (fetal bovine serum), 5.5 mM D-glucose, 10 ng/ml human recombinant epidermal growth factor, and 750 ng/ml puromycin. MIA PaCa-2 cells were cultured in DMEM, supplemented with 10% FBS and 2.5% horse serum. The remaining cell lines were maintained in the respective media suggested by the ATCC (DMEM, RPMI-1640, Eagle's Minimum Essential Medium, and Iscove's DMEM) supplemented with 10% FBS, except for Panc 10.05 that required 15% FBS. All media contained 100 U/ml penicillin, and 100 U/ml streptomycin. All media for the above cell lines were purchased from Mediatech (Manassas, VA, U.S.A.) whereas FBS was from Atlanta Biologicals (Atlanta, GA, U.S.A.), and plasticware for cell culture was obtained from Corning LifeSciences (Manassas, VA, U.S.A.). Cells were cultured at 37°C in a humidified atmosphere containing 5% CO_2_. All these cell lines have been routinely tested for mycoplasma contamination using the Universal Mycoplasma Detection Kit obtained from ATCC.

### RNA isolation and Real-time RT-qPCR

RNA isolation and Real-time RT-qPCR were performed as described previously [29]. Briefly, RNA was isolated from cells using Trizol method. The expression of the genes was analyzed using real-time RT-qPCR. RNA samples from normal pancreatic tissues and pancreatic tumor tissues were obtained from Asterand (Detroit, MI, U.S.A.). After isolation, RNA concentration was measured using a Nanodrop ND-1000 system, followed by DNase treatment with DNase kit (Promega, Madison, WI, U.S.A.) and then the cDNA was synthesized using high capacity cDNA synthesis kit (Invitrogen, Grand Island, NY, U.S.A.). Relative mRNA levels were measured with SYBR Green detection system using the QuantStudio3 real-time PCR system (Applied Biosystems, Foster City, CA, U.S.A.). The samples were used in triplicates and the relative level of each gene was calculated by normalizing the cycle threshold (Ct) value of the gene being studied to that of the housekeeping gene GAPDH. The following primers were used (IDO1; forward: 5′-GAGGAGCAGACTACAAGAATGG-3′, reverse: 5′-GTGGATTTGGCAGAGCAAAG-3′), (STAT1; forward: 5′-AGGCCAAAGGAAGCACCAGAG-3′, reverse: 5′-GCAGGTTGTCTGTGGTCTGAA-3′), and (CYP1A1; forward: 5′-CAAGGGGCGTTGTGTCTT TG-3′, reverse: 5′-GTCGATAGCACCATCAGGGG-3′).

### Western blot and immunoprecipitation

Western blotting was performed as described previously [56,63]. Briefly, cells were grown to 80–90% confluency, and following treatment with carbidopa, whole cell lysates were prepared in RIPA lysis buffer with a protease inhibitor cocktail and a phosphatase inhibitor cocktail. An amount of 20 µg of protein was loaded per lane in each of the western blots and electrophoretically separated on 10% SDS–Page gels and all proteins were transferred at 20 V overnight at 4°C to a nitrocellulose membrane (Bio-Rad, Hercules, CA, U.S.A.). The membrane was then briefly washed and blocked with 5% Blotting Grade Blocker (Bio-Rad) at room temperature for 1 h. The blocked membrane was incubated with a primary antibody overnight at 4°C and washed three times with TBST for 10 min each. The membrane was incubated for 1 h at room temperature with correlating secondary antibody and washed as before. The membrane was then incubated for 5 min in Pierce ECL solution (ThermoFisher, Waltham, MA, U.S.A.), before being visualized with autoradiography films. For immunoprecipitation experiments, cells were first cultured in the presence and absence of increasing concentrations of Carbidopa, followed by immunoprecipitation with either anti-AhR or anti-STAT1 antibody as well as rabbit IgG isotype control and then subjected to SDS–PAGE.

### Chromatin immunoprecipitation assay

Chromatin immunoprecipitation (ChIP) assay was performed using a simpleChIP Enzymatic Chromatin IP Kit (Cell Signaling Technology, U.S.A.) according to the manufacturer's protocol. The IDO1 promoter primers used were: (forward) 5′-GTC CTG ATT AAA TGG GTA CCA GA-3′ and (reverse) 5′-GTC TAG AAC CAT GTT CCG AAG C-3′. HPAF-II cells were seeded at 1.0 × 10^6^ cells in a 10 cm dish. After 2 days, the cells were treated with Carbidopa (50 & 100 µM) for 3 h. DNA–protein complexes were cross-linked with 1% formaldehyde for 10 min at room temperature. The cross-linking reaction was stopped by addition of 1 ml of 10× glycine and incubated for 5 min at room temperature, cells were washed with ice-cold PBS and harvested in ice-cold PBS containing protease inhibitors provided with the kit. Cells were centrifuged and the DNA was digested with Micrococcal Nuclease at 37°C for 20 min with frequent mixing. The reaction was stopped by addition of 0.5 M EDTA and incubating on ice for 2 min. The nuclei were pelleted by centrifugation at 16 000×***g*** for 1 min and resuspended in 1× Chip buffer and incubated for 10 min on ice. The lysates were sonicated for three cycles (20 s ON, 30 s OFF) on ice to break the nuclear membrane. The lysates were clarified by centrifuging the samples at 9400×***g*** for 10 min at 4°C. The chromatin digestion was cross-linked and concentrated. DNA was purified using DNA purification columns. 10 µg of chromatin digest and cross-linked sample was used for each IP in 500 µl Chip buffer and 10 µl of sample was taken for 2% input. The prepared chromatin digest was incubated with AhR antibody (1 : 100) overnight at 4°C. The sample were added with 30 µl of protein G Magnetic bead and incubated at 4°C for 2 h. The protein G magnetic bead was pelleted in magnetic separation rack. After the bound chromatin was washed three times with low salt washes followed by one high salt wash, the chromatin was eluted from the antibody/protein G magnetic beads for 30 min at 65°C with gentle vertexing. The protein G magnetic bead were pelleted and the eluted chromatin was carefully transfer. The DNA–protein cross-links were reversed using proteinase K and 5 M NaCl solution by incubation at 65°C for 2 h. The AhR-enriched fraction of genomic DNA was purified. PCR was used to analyze ChIP DNA for enrichment at a region on the IDO1 promoters. The DNA polymerase was heat-activated at 95°C for 5 min followed by 40 cycles, denaturing at 95°C for 30 s, annealing at 62°C for 30 s, elongating at 72°C for 30 s and final extension at 72°C for 5 min. PCR products were separated on 1.5% agarose gels and visualized by ethidium bromide staining.

### Xenograft studies

Xenograft studies were performed as described previously [29,62]. Eight-week-old female athymic nude mice, purchased from Jackson Laboratories, were allowed to acclimatize to the environment for about a week before the start of the experiment. Mice were injected with 1 × 10^6^ of HPAF-II and 8 × 10^6^ of BxPC-3 cells subcutaneously into each flank. All cells were suspended in serum-free media and Matrigel (1 : 1 ratio), with 100 µl of suspension being injected subcutaneously in each mouse. Mice were randomly divided into two/four groups — control and treatment. Control group received regular drinking water whereas the treatment group received 1 mg/ml of Carbidopa in their drinking water or 50 mg/kg of gemcitabine intraperitoneally once a week. In another set of experiment, carbidopa was administered to the mice @ 1 mg/200 µl of canola oil suspension once a day through oral gavage. Same volume of canola oil was administered through oral gavage to the control mice. Tumor size was measured using a caliper three times a week, and the tumor volume was calculated using the formula (width^2^ × length)/2. The experiment was terminated 20–45 days post injection; mice were killed via isoflurane induction and tumors harvested following the approved IACUC protocol. RNA was prepared from the tumor tissue for RT-qPCR to determine the expression levels of IDO1 in both control and carbidopa-treated groups. All murine experiments were performed in the Laboratory Animal Resource Center at Texas Tech University Health Science Center in Lubbock, TX.

### Statistical analysis

Statistical analysis and graphs were performed using GraphPad Prism 8.4.3. Results are expressed as mean ± SEM. All experiments were repeated thrice unless otherwise specified. Statistical significance was determined using either unpaired Student's *t*-test or one-way analysis of variance (ANOVA) with a Dunnett's or Tukey's post hoc test for multiple comparisons, and *P* values have been indicated as follows: *** P < 0.001; ** *P* < 0.01; * *P* < 0.05; ns > 0.05.

## Data Availability

All datasets generated and/or analyzed during the current study are included in this manuscript and in the supplementary information files.

## Open Access

Open access for this article was enabled by the participation of Texas Tech University Health Sciences in an all-inclusive *Read & Publish* agreement with Portland Press and the Biochemical Society Center.

## Abbreviations

AhR: Aryl hydrocarbon receptor
CYP1A1: Cytochrome P450 family 1 subfamily A member 1
D-1MT: 1-methyl-D-tryptophan
DC: Dendritic cells
GCN2: General control nonrepressible 2
IDO1: Indoleamine-2,3-dioxygenase 1
JAK: Janus kinase
L-DOPA: L-3,4-Dihydroxyphenylalanine
mTORC1: Mechanistic target of rapamycin 1
PDAC: Pancreatic ductal adenocarcinoma
STAT: Signal transducer and activator of transcription
TCDD: 2,3,7,8-tetrachlorodibenzo-p-dioxin
TME: Tumor microenvironment
Trp: Tryptophan
