# Supplementary material for: Carbidopa, an activator of aryl hydrocarbon receptor, suppresses IDO1 expression in pancreatic cancer and decreases tumor growth
Source: Biochem J. 2022 Sep 6;479(17):1807–24. doi: 10.1042/BCJ20210851 (PMC9472820; doi:10.1042/BCJ20210851)
Supplement: Supplementary Material [file BCJ-479-1807-s1.pdf]

Supplemental material

Figure legend

Figure S1. Carbidopa-mediated IDO1 suppression attenuates tumor growth in athymic nude mice. BxPC-3 and HPAF-II cells were subcutaneously implanted in athymic nude mice and administered carbidopa via oral gavage. Representative photographs of harvested tumors from control and carbidopa-treated mice bearing BxPC-3 cells (**A**) and HPAF-II cells (**E**). Tumor weights between control and carbidopa-treated mice bearing BxPC-3 (**B**) and HPAF-II cells (**F**). Evaluation of mouse body weights during the xenograft experiments: BxPC-3 (**C**); HPAF-II (**G**). Real-Time PCR showing relative IDO1 mRNA expression in control and carbidopa-treated BxPC-3 (**D**) and HPAF-II (**H**) tumor xenograft samples extracted from the athymic nude mice. Data are given as mean ± SEM. $*p<0.05$, $***p<0.001$.

Figure S2 ~~S1~~. Carbidopa does not synergize with gemcitabine but is potent by itself in attenuating tumor growth in athymic nude mice. BxPC-3 cells were subcutaneously implanted in athymic nude mice. **A**. Tumor growth curves from control and drug-treated (Carbidopa, gemcitabine, and Carbidopa + gemcitabine) mice. **B.** Tumor weights between control and drug-treated mice. Data are given as mean ± SEM. *p<0.05; ***p<0.001.

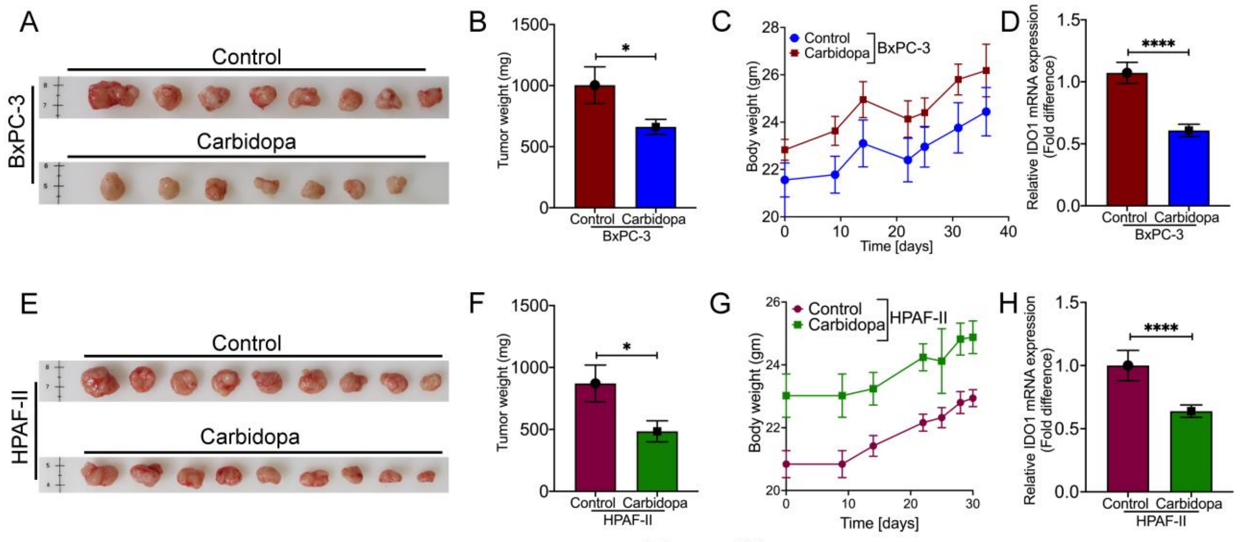

A
Control
Carbidopa
BxPC-3
B
Tumor weight (mg)
1500
1000
500
0
*
Control Carbidopa
BxPC-3
C
Body weight (gm)
28
26
24
22
20
Control
Carbidopa
BxPC-3
0 10 20 30 40
Time [days]
D
Relative IDO1 mRNA expression (Fold difference)
1.5
1.0
0.5
0.0
****
Control Carbidopa
BxPC-3
E
Control
Carbidopa
HPAF-II
F
Tumor weight (mg)
1500
1000
500
0
*
Control Carbidopa
HPAF-II
G
Body weight (gm)
26
24
22
20
Control
Carbidopa
HPAF-II
0 10 20 30
Time [days]
H
Relative IDO1 mRNA expression (Fold difference)
1.5
1.0
0.5
0.0
****
Control Carbidopa
HPAF-II

Figure S1

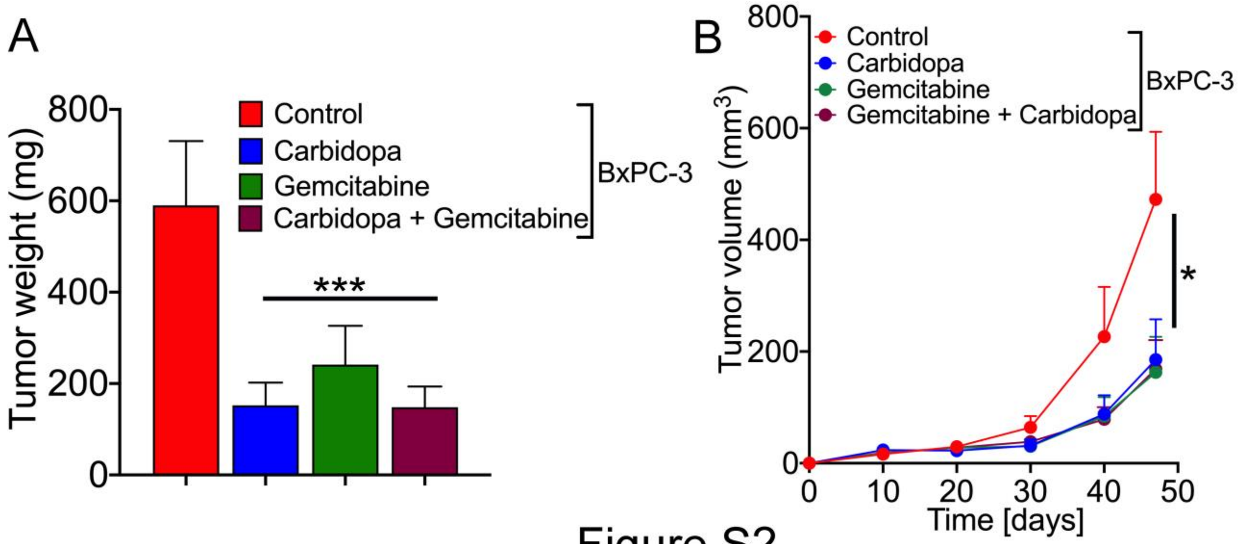
A
Tumor weight (mg)
800
600
400
200
0
Control
Carbidopa
Gemcitabine
Carbidopa + Gemcitabine
BxPC-3
***
B
Tumor volume (mm3)
800
600
400
200
0
Control
Carbidopa
Gemcitabine
Gemcitabine + Carbidopa
BxPC-3
*
0
10
20
30
40
50
Time [days]

Figure S2
